# Cross-Layer Active Predictive Congestion Control Protocol for Wireless Sensor Networks

**DOI:** 10.3390/s91008278

**Published:** 2009-10-20

**Authors:** Jiangwen Wan, Xiaofeng Xu, Renjian Feng, Yinfeng Wu

**Affiliations:** 1 School of Instrument Science and Opto-electronics Engineering, Beijing University of Aeronautics and Astronautics (Beihang University), Beijing 100191, China; E-Mails: rjfeng@buaa.edu.cn (R.J.F.); yfwu@buaa.edu.cn (Y.F.W.); 2 School of Computer Science and Technology, Beijing University of Posts and Telecommunications, Beijing 100876, China; E-Mail: alickelly@gamil.com (X.F.X.)

**Keywords:** wireless sensor networks, congestion control, dynamic priority, cross-layer protocol

## Abstract

In wireless sensor networks (WSNs), there are numerous factors that may cause network congestion problems, such as the many-to-one communication modes, mutual interference of wireless links, dynamic changes of network topology and the memory-restrained characteristics of nodes. All these factors result in a network being more vulnerable to congestion. In this paper, a cross-layer active predictive congestion control scheme (CL-APCC) for improving the performance of networks is proposed. Queuing theory is applied in the CL-APCC to analyze data flows of a single-node according to its memory status, combined with the analysis of the average occupied memory size of local networks. It also analyzes the current data change trends of local networks to forecast and actively adjust the sending rate of the node in the next period. In order to ensure the fairness and timeliness of the network, the IEEE 802.11 protocol is revised based on waiting time, the number of the node's neighbors and the original priority of data packets, which dynamically adjusts the sending priority of the node. The performance of CL-APCC, which is evaluated by extensive simulation experiments. is more efficient in solving the congestion in WSNs. Furthermore, it is clear that the proposed scheme has an outstanding advantage in terms of improving the fairness and lifetime of networks.

## 1. Introduction

Recent technology development in the fields of wireless communication and MEMS has made extensive distribution of wireless sensor networks (WSNs) become possible. It is obvious that WSNs are reliable, accurate, flexible, inexpensive, easy to deploy and have other excellent features. As such, they have potential applications in many areas. For instance, monitoring is one of the most important applications of WSNs, such as the monitoring of agricultural crops, buildings, water quality, etc. These types of sensor networks have the characteristics of centralized data collection, multi-hop data transmission, many-to-one flow patterns, as well as increased data flow brought about by contingency. The above characteristics easily lead to the part or overall congestion of WSNs, which seriously influences the quality of the service of networks [1]. This can include increased delays in transmitting information and the loss of data packets. It also leads to repeated data sending that further increases the flow of the network, which wastes valuable energy, bandwidth and other network resources.

A traditional wired network [2] merely affords a transmission platform for data. It adheres to the end-to-end design concept and the intermediate nodes are only responsible for retransmitting data. However, WSNs are different from wired networks in that they are data-centered networks and the intermediate nodes also process data packet; in addition, the physical equipment of the nodes is often subject to destruction and has limited energy. Also, the wireless channel is vulnerable to be interfered with by other transmission signals. All the characteristics mentioned above increase the difficulty of controlling the congestion of WSNs. Therefore, the traditional network congestion control schemes, such as TCP, UDP, etc. cannot meet the requirements of WSNs, which makes the research work on congestion for WSNs more significant and challenging.

Consequently, it is necessary to efficiently control the data transmission of WSNs, which aims at avoiding or properly relieving the occurrence of network's congestion. To be integrated with network congestion and fairness, a cross-layer active predictive congestion control scheme is proposed, which is based on the occupied node memory and data flow trends of local network (grid), as well as combined with network conditions and node rate within period *t*. It aims at predicting the inputting and outputting rates of node within the next period *t* + 1 in order to avoid the congestion. The fairness of network and the timeliness of data packets are also taken into account by the design of cross-layer scheme.

The remainder of this paper is organized as follows. Section 2 introduces the related work including typical congestion control protocols. Section 3 provides system architecture and basic models of WSN for later analysis. In Section 4, the proposed scheme is presented in detail, which includes control methods of congestion in node-level and system-level, as well as the revised IEEE 802.11 protocol. Section 5 does the specific analysis based on each performance of CL-APCC. The performance of CL-APCC is mainly evaluated in Section 6. Finally, in Section 7, we make some concluding remarks and outline some future work.

## 2. Related Work

The growing interest in WSNs and the continual emergence of new techniques has inspired some efforts to design congestion control protocols in this area. Normally, current congestion control protocols in WSNs can be typically classified into two broad categories [3-34]: congestion avoidance mechanisms and congestion release mechanisms [10-14]. (1) The existing congestion avoidance mechanism is mainly achieved by two kinds of methods. ➀ The first is the rate allocation method [10,11]. It requires that the sending rate allocated to the node must be equal to the sum rates which are the rate of generated data by the node and that of all its children nodes. But it is very difficult to allocate the sending rate for each node under the dynamic condition of network topology. Furthermore, if some nodes in the network are not active, bandwidth and other resources of network will be wasted. ➁ The second is the cache notification method [12-14] which is passively applied to the circumstances where network congestion has already occurred. In addition, this method can effectively avoid the phenomenon of node-level congestion, however, the system-level congestion (local network congestion) is inevitable. (2) Congestion release mechanism [15-34] is achieved by methods of rate adjustment. The weakness of this mechanism is the same as ➁.

Although the two categories above are good for improving network performance, nevertheless, all such methods like (1) and (2) are passive adjustments after the occurrence of congestion (energy and bandwidth have been wasted). In addition, more attention should be paid to address the issues of QoS of network.

The Congestion Control and Fairness (CCF) routing scheme [10] uses packet service time at the node as an indicator of congestion. However, the service time alone may be misleading when the incoming rate is equal or lower than the outgoing rate through the channel with high utilization. On the other hand, the Priority-based Congestion Control Protocol (PCCP) [11] rectifies this deficiency by observing the ratio between packet service time and inter-arrival time at a given node to assess the congestion level. However, both CCF and PCCP ignore current queue utilization which leads to increased queuing delays and frequent buffer overflows accompanied by increased retransmissions.

The CODA protocol [12] uses both a hop-by-hop and an end-to-end congestion control scheme to react to the congestion by simply dropping packets at the node preceding the congestion area and employing the additive increase and multiplicative decrease (AIMD) scheme to control a source's generation rate. Thus, CODA partially minimizes the effects of congestion, and as a result retransmissions still occur. Similar to CODA, Fusion [13] uses a static threshold value for detecting the onset of congestion even though it is normally difficult to determine a suitable threshold value that works in dynamic channel environments. In both CODA and Fusion protocols, nodes use a broadcast message to inform their neighboring nodes the onset of congestion, though this message is not guaranteed to reach the sources.

The interference-aware fair rate control (IFRC) protocol [14] uses static queue thresholds to determine congestion levels, whereas IFRC exercises congestion control by adjusting the outgoing rate on each link based on the AIMD scheme. Consequently, the IFRC reduces the number of dropped packets by reducing the throughput. By contrast, the proposed scheme varies the rate adoptively based on the current and predicted congestion level. The control parameters in the proposed scheme are updated according to changing environment, while the IFRC [14] and others [12,13] require that the parameters and thresholds have to be selected before each network deployment.

SenTCP [15] is an open-loop hop-by-hop congestion control with a few special features. It jointly uses the average local packets service time and the average local packet inter-arrival time to estimate the current local congestion degree in each intermediate node. During congestion it uses hop-by-hop congestion control. But, SenTCP needs strict time synchronization between nodes, which is difficult for WSNs.

In the Event-to-Sink Reliable Transport (ESRT) protocol [16], a sensor sets a congestion-notification (CN) bit in the packet header if its buffer is about full, which is like the (RT)^2^ protocol [17]. The sink periodically computes a new reporting rate (at which each source is supposed to report data) based on a reliability measurement, the received CN bits, and the previous reporting rate. It then broadcasts the new reporting rate to all data sources. Treating all sources equally is suboptimal. To remove all congestions, the reporting rate has to be set according to the worst hotspot in the network. In that case, the noncongested sources will be constrained by a conservative reporting rate.

As the communication cost from different sources to the sink may be different and may change dynamically, and the contributions of packets from different sources are also different, it is necessary to bias the reporting rates of the sources. But, ESRT adjusts the report rate of sources in an undifferentiated manner. Based on these defects of ESRT, Price-Oriented Reliable Transport protocol (PORT) [18] employs node price, which is defined as the total number of transmission attempts across the network needed to achieve successful packet delivery from a node to the sink to measure the communication cost. At the same time, PORT dynamically feeds back the optimal reporting rate to each source according to the current contribution of the packets from each source and the node price of each source.

In addition, other protocols [19-34] address congestion control from different angles too, but the related works proposed above adopt a passive approach to congestion adjustment. When congestion has occurred in the network, data source control or flow distribution is carried out passively through the feedback. At this point, a lot of useless information has been sent in the network, which results in wasted bandwidth and sensor energy. Naturally, The Active Congestion Control methods [35,36] have become particularly important in order to reduce energy overhead and improve bandwidth utilization ratios.

Although the existing schemes play important roles to improve network performance, congestion control is still a challenging area in WSNs. In this paper, an active predictive method based on the research of other relevant protocols is demonstrated. The proposed scheme utilizes the priority of data sending to adjust the inputting and outputting rates of nodes, which prevents the occurrence of network congestion and ensures the timeliness and fairness of data transmission. The difference in our work from the aforementioned approaches mainly includes the following three aspects: (1) In order to actively predict and solve single-node congestion in networks, a single service window with the mixed queuing model M/M/1/m is applied to deal with the issue. (2) The predictive method of periodic flow is adopted to solve the congestion of local networks (grid). (3) The IEEE 802.11 protocol is revised to ensure fairness and timeliness of data transmission in the network by the design of cross-layer (according to the original priority and waiting time of data packets to make sending strategy).

## 3. Preliminaries

In this section, we describe the system architecture of congestion control protocol and various parameter variable definitions in WSNs. The overall network is divided into grids to better control the congestion of the network. In each grid, the predictive periodic flow method is used to solve the congestion. The basic grid models are illustrated for later analysis, including definition of each period length, selection of node with flow predictive feature (named A-type node) and the status of how to deal with failure of an A-type node in each grid.

### 3.1. System Architecture

**Figure 1.**
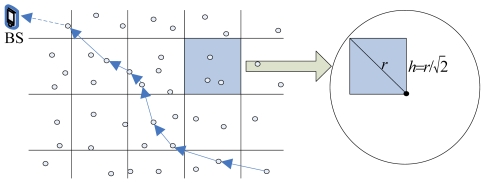
Grid definition.

Here, we consider a scenario where the WSN is formed by stationary sensors in a two-dimension ***R**^2^* sensing field. In order to better control congestion of local network, there are several main parameters to be set. The whole of ***R**^2^* space is divided into the grids number, *K*, and each grid is deployed in a square with the length of side, *h*. So the grid number *K* = (*H* × *H*)/(*h* × *h*), the value of *k* increases in turn from left to right, and the grid value of the lower level is often bigger than that of the upper level (as shown in the Figure 1). Nodes are intensive enough and randomly distributed. The initial energy of each node is homogeneous and can not be complemented. The communication radius of the node is *r*, and the aim of $r\geq \sqrt{2}h$ is to ensure each node in the same grid can normally communicate. Each node knows its initial position. Sensors periodically report the information about the monitored events to BS. For each node, any other node in its communication range could become its neighbor.

In order to further to demonstrate the node and packets of the network, it is necessary to demonstrate the node *i*. The coordinates of *i* are *v*(*X*_i_, *Y*_i_), so according to the initial position, the grid value of the node *i* is *k_i_* = [*Y_i_/h*] × (*H/h* − 1) + [*X_i_/h*], and the number of *i*'s neighboring node is $n_{\text{con}}^{i}$. The length of the data packet is $L_{\text{Data}}^{q}$. The packet header structure of node *i* in grid *k* is: $PH_{i}(t)=PH_{i}(t)=\left\{\left(k_{i},{\text{ParentIDs}}_{i},{\text{ChildIDs}}_{i},\lambda_{i,}^{k}(t),\mu_{i}^{k}(t),\lambda_{\text{avg}}^{k}(t),\mu_{\text{avg}}^{k}(t),En_{i}(t)\right)|i\in R^{2}\right\}$, among which the variable of *ParentIDS*_i_ is a direct upstream neighboring set of node *i* (near to the BS) and the variable of *ChildIDS*_i_ which is a downstream neighboring set of node *i* (around the data sources). These two variables are decided by the specific routing algorithms. $\lambda_{i}^{k}(t)$ and $\mu_{i}^{k}(t)$ which respectively represent the inputting and outputting rate of node *i* in period *t*. $\lambda_{\text{avg}}^{k}(t)$ and $\mu_{\text{avg}}^{k}(t)$ are separately expressed average inputting and outputting rate of grid *k*, and the last variable of *En_i_*(*t*) represents the current residual energy of node *i*.

### 3.2. The Grid Structure

In order to better control congestion of a local network, the whole network is divided into grids. In each grid, the predictive method of periodic flow is adopted to solve local congestion. The detailed demonstration of the grid structure is illustrated below.

First, we explain how to select the A-type in each grid. A node is randomly selected as the A-type node in each grid during the initialization of the network. In the operation process of the system, the packet header of the node has its own residual energy information *En_i_*(*t*) (we describe the packet header structure of the node in subsection 3.1). In order to better explain this issue, we assume that the current time of grid *k* is in period *t* and the A-type node is *A*_k_(*t*). Residual energy *En_i_*(*t*) of node *i* in the packet header was monitored by *A*_k_(*t*). At the end of period *t*, a new A-type node (*A*_k_(*t* + 1)) from the next period *t* + 1 is changed into the node which has the maximum residual energy (Max(*En_i_*(*t*)) in the grid. At the same time, *A*_k_(*t*) broadcasts the new A-type node (*A*_k_(*t* + 1)) to all nodes in the grid.

Second, the period of each grid is described. The A-type node has been set to promiscuous mode to monitor all nodes in the grid. Once the A-type node has monitored that all nodes in the grid have already sent data one time, then it broadcasts to the other nodes in the grid that this period is finished and notices which node becomes a new A-type node in the next period. Concurrently, a new period begins and the new A-type node starts to monitor the data flow of the grid under promiscuous mode in the new period.

Third, it is illustrated what to do if the A-type node fails. The period of each grid has its own maximum time *Max*(*t*) (*Max*(*t*) is described in subsection 5.1). Hence, each node maintains a counter to reflect the broadcast signal of the A-type node in the grid. If the node doesn't receive the broadcast signal from the A-type before the maximum time of its own grid, then the node believes that the A-type node has failed. The data sending sequence of the node which ranks first place in the grid (there will be illumination about the data sending sequence of the nodes in Section 4.2) will temporarily be used as the A-type node. Concurrently, the temporary A-type node broadcasts itself to all nodes in the grid. In the next period, the A-type node is still chosen to be the node which has the maximum residual energy in the grid.

## 4. CL-APCC Protocol

Congestion is mainly caused by two factors. First, data is received too quickly, which leads to the overflow of the node. Second, a great deal of collisions occur between data packets which are sent by nodes. Hence, according to periodicity reports or data flow characteristics of application types such as emergency monitoring, we separately analyze the existence probability of node-level congestion and local congestion(system-level) in the network in order to overcome the node overflow. The probability of congestion is analyzed to dynamically adjust the receiving and sending rate of nodes. Consequently, the IEEE 802.11 protocol is revised, which is designed to reduce the collision probability of packets and guarantee the fairness of network transmission. Normally, CL-APCC is divided into two steps. (1) According to the node memory size, the scheme adopts a single service window of the mixed queuing model M/M/1/m to predict and solve the node-level congestion. Then, according to the average inputting and outputting rate of the grid and the status of occupied node's memory size in the period *t* – 1, the CL-APCC predicts the grid's probability of congestion within the period *t*. In this way it controls the average inputting and outputting rate of the grid based on the probability of congestion. This solution is used to solve the congestion of local networks (system-level). Finally, according to the adjustment method of node-level and system-level, the real sending rate of node is set to solve the congestion of the entire network. (2) The IEEE 802.11 protocol is revised in the foundation of the first step, which is aimed to guarantee the fairness of network and the timeliness of data packets. The design principle of this protocol is described below.

**Figure 2.**
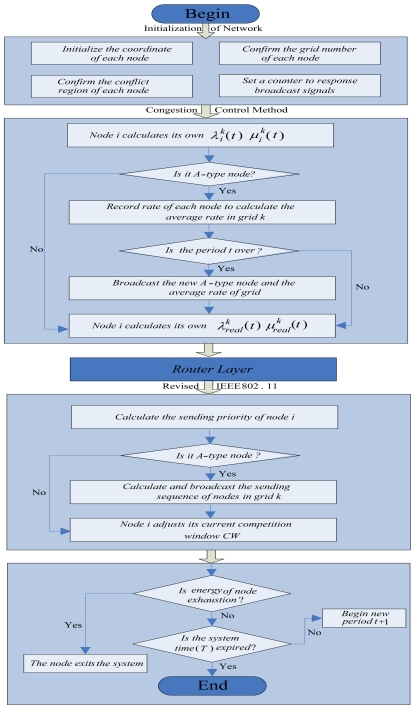
The data flow in the node.

We adopt the modular mechanism from the top to bottom to describe the data flow in any node. Node *i* is analyzed in a certain grid *k*. Firstly, it is an initialization module which includes the initialization of the node coordinate, the grid number of the node, the conflict region of the node and the initial rate of the node. Secondly, it is a congestion control module. In this module, node *i* first calculates its own inputting rate $\lambda_{i}^{k}(t)$ and outputting rate $\mu_{i}^{k}(t)$ in period *t* according to the node-level congestion control method. Next, if node *i* is not an A-type node, it is necessary that node *i* immediately calculates its real rate $\lambda_{\text{real},i}^{k}(t)$ and $\mu_{\text{real},i}^{k}(t)$. On the contrary, if node *i* is the A-type node, it records the rate of each node in grid *k* for the calculation of the average rate in period *t* + 1. At the end of period *t*, node *i* broadcasts what is the new A-type node and the average rate of grid *k*. Thirdly, data stream flows into a routing layer and from there it flows into the lower layer (the specific routing layer is not taken into account in this manuscript). Fourthly, the data stream flows into the revised IEEE 802.11 module. In this module, at first, it is calculated in the first slot for the sending priority of node *i*. Then it is analyzed whether or not the node is an A-type node. If so, node *i* records and broadcasts the sending sequences of all nodes in grid *k*. On the contrary, if node *i* is not the A-type node, with the increase of times that node *i* competes for channels, the CW gradually decreases, which gives the node a higher probability of acquiring the current slot. The final module is the one which is determined by nodes whether or not they exit the system (see Figure 2 for the data flow in any node)

### 4.1. Network Congestion Control Methods

Congestion has local relevance. That is, congestion would normally appear in a number of nodes in the local network. Furthermore, the control method of node-level congestion cannot entirely reflect the real status of a network. Therefore, CL-APCC respectively analyzes a single node and the data flow of the grid where the node lies. Based on the analysis, the rate control method is adopted in advance to avoid congestion occurring.

Here, a simple description is made to introduce the congestion method of the node-level and system-level. Assume that the occupied node memory size is *L*, the maximum node memory size is *m*, and threshold value is *L*_max_ [15]. Firstly, we describe the congestion control method of the node-level. If the occupied node memory size is *L* < *L*_max_, CL-APCC identifies the node isn't congested. If the memory size is *L*_max_ < *L* < *m*, then CL-APCC identifies the congestion of node-level to have probably occurred. In this case, the sending rate of the node is adjusted to control the congestion. If the occupied node memory size reaches the maximum(*L* = *m*), the input rate of the node is adjusted to 0. Secondly, we describe the congestion control method of the system-level. At the beginning of a new period *t*, CL-APCC protocol makes a prediction for the expected value *E*(t) of data quantity in period *t* using the average rate in period *t* – 1 in the grid. If *E*(*t*) < *n* × *L*_max_(*n* is the number of nodes in the grid), congestion of system-level does not occur. On the contrary, if *n* × *m* > *E*(*t*) > *n* × *L*_max_, congestion of system-level may occur. In this case, the average inputting and outputting rates of the network are adjusted to avoid system-level congestion (because we strictly control the margin between the inputting and outputting rate, it could not occur *E*(t) > *n* × *m*).

**Figure 3.**
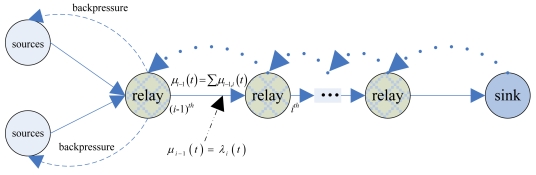
The relationship between inputting and outputting rate of node.

In practical applications, according to the status of network resource, CL-APCC separately sets a weight for node-level and system-level to obtain the real rate of each node. Assume that the total outputting rate of the upper nodes is equal to the inputting rate of the lower nodes. This means that the outputting rate of node *i* is decided by the inputting rate of node *i* + 1. Each node obtains its data retransmission rate according to the relationship between inputting and outputting (the model of the sending rate is shown in Figure 3).

Now, the congestion methods of the node-level and system-level are described in detail below. Node *i* is analyzed in a certain grid *k*.

#### 4.1.1. The pre-control and adjustment method of node-level rate

**Figure 4.**
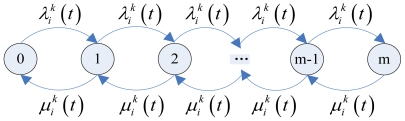
The flow chart of the stable state M/M/1/m.

It is better to adopt a single service window of the mixed queuing model M/M/1/m [38] to make an analysis of the inputting and outputting rate of each node because of the limited memory size. The stable state of the queuing model is shown in Figure 4.

It can be seen from Figure 4, the stable state model Equation as follows:

(1) $$\begin{array}{c} \lambda_{i}^{k}(t)\times p_{0}=\mu_{i}^{k}(t)\times p_{1}\Rightarrow p_{1}=\lambda_{i}^{k}(t)/\mu_{i}^{k}(t)p_{0}=\rho p_{0}\;\rho =\lambda_{i}^{k}(t)/\mu_{i}^{k}(t)\\ \lambda_{i}^{k}(t)\times p_{1}=\mu_{i}^{k}(t)\times p_{2}\Rightarrow p_{2}=\lambda_{i}^{k}(t)/\mu_{i}^{k}(t)p_{1}=\rho^{2}p_{0}\\ \begin{array}{cccccc} \cdot & \cdot & \cdot & \cdot & \cdot & \cdot \end{array}\\ \lambda_{i}^{k}(t)\times p_{m-1}=\mu_{i}^{k}(t)\times p_{m}\Rightarrow p_{m}=\lambda_{i}^{k}(t)/\mu_{i}^{k}(t)p_{m-1}=\rho^{m}p_{0} \end{array}$$

By the regularity conditions reduced.

(2) $$1=\sum_{\eta =0}^{m}p_{n}=\sum_{\eta =0}^{m}\rho^{\eta }p_{0}=\frac{1-\rho^{m+1}}{1-\rho }\times p_{0}$$

The conclusion is:

(3) $$\begin{array}{c} p_{0}=\frac{1-\rho }{1-\rho^{m+1}}\\ \begin{array}{cccccc} \cdot & \cdot & \cdot & \cdot & \cdot & \cdot \end{array}\\ p_{L}=\frac{1-\rho }{1-\rho^{m+1}}\times \rho^{L}\;L=1,2,\ldots ,m \end{array}$$

According to the analysis of telecommunications network model [39,40], if *ρ* ≤ 0.6, the quantities of data packets are slowly increased in the network, and then the system gradually achieves the optimal state. If *ρ* > 0.6, the system quickly reaches the saturation state which leads to data packets badly overflowing. Thus, the sample rates of data sources based on data requirements of the BS in unit time can be obtained as well as the constraints of *ρ* value. Also, from literature [15], the node may appear congested in the WSNs if a node's occupied memory size is larger than a threshold *L*_max_. Therefore, the total congestion probability of the node occurred as follows:

(4) $$P_{\text{con}}=p_{L_{\max}}+p_{L_{\max+1}}+p_{L_{\max+2}}\cdots +p_{m}$$

namely:

(5) $$P_{\text{con}}=\sum_{\text{L}={\text{L}}_{\max}}^{\text{m}}p_{L}=\sum_{\text{L}={\text{L}}_{\max}}^{\text{m}}\left(\frac{1-\rho }{1-\rho^{m+1}}\times \rho^{L}\right)$$

From Equation (5), we can see the relationship between *ρ*, occupied node memory size *L* and the congestion probability of the node. Thus, the retransmission rate of intermediate nodes can be obtained according to the data quantity requirements of BS.

Assume that occupied node memory size is *L* (*m* > *L* > *L*_max_), therefore, its congestion probability is as follows:

(6) $$p_{\max}^{\text{con}}=\frac{\left(m-L_{\max})-(m-L\right)}{m-L_{\max}}=1-\frac{m-L}{m-L_{\max}}$$

To correspondingly reduce the value of *ρ*:

(7) $$\rho =\left(1-p_{\max}^{\text{con}}\right)\rho =\left(1-1+\frac{m-L}{m-L_{\max}}\right)\times \rho \frac{m-L}{m-L_{\max}}\times \rho$$

We discuss the changing status of the node's rate from two aspects based on the adjustment of *ρ*. ➀ After some time, if the occupied node memory size *L* regains access to the optimal interval, namely *L* ∈ [0,*L*_max_], then the value of *ρ* is maintained unchanged (If *ρ* is adjusted to 0.6, this leads the node back to the state of congestion and consequently cause frequently oscillating). ➁ If *L* = *m*, the inputting rate of node *i* is set to be 0, namely $\lambda_{i}^{k}(t)=0$. That is because the channel from node *i* to *i* + 1 may experience large scale fading (the period length of large scale fading is $\left\lceil 2L_{\text{Data}}^{q}/\mu_{k}^{i}(t)\right\rceil$, the further detail to explain this issue has been discussed in the next paragraph), it is necessary for node *i* to lose packets, so CL-APCC protocol sets node *i* to lose the data packet which has the lowest priority (we will discuss the issue of priority in subsection 4.2).

Node *i* notifies node *i* – 1 through an ACK message, and then node *i* – 1 sends feedback “ACK” to node *i* – 2 and iterates it to data sources which correspondingly reduce the sampling frequency. Node *i* is trying to send data after a random period of time (the length of random time is set to be the value between $\left\lceil 2L_{\text{Data}}^{q}/\mu_{\text{con}}^{i}(t)\right\rceil$ and $\left\lceil 3L_{\text{Data}}^{q}/\mu_{\text{con}}^{i}(t)\right\rceil$, which is decided by the period length of large scale fading.). If possible, node *i* first send data at the maximum rate. If the occupied node memory size regains access to the optimal interval, then the node *i* adjusts *ρ* = 0.6. Meanwhile, node *i* notifies node *i* – 1 using ACK message and the message is iterated to the data sources.

Here, we describe the reason that the period length of large scale fading is not $\left\lceil L_{\text{Data}}^{q}/\mu_{k}^{i}(t)\right\rceil$ but $\left\lceil 2L_{\text{Data}}^{q}/\mu_{k}^{i}(t)\right\rceil$. In the network, it is seen that the earlier large scale fading is found, the better the results. Because it can not only save the energy of network, but also avoid the waste of bandwidth. Under ideal conditions, it is better that the large scale fading can be found once it has occurred. So, it seems that the period length of large scale fading should be $\left\lceil L_{\text{Data}}^{q}/\mu_{k}^{i}(t)\right\rceil$. Nevertheless, we set the period length of large scale fading is not at $\left\lceil L_{\text{Data}}^{q}/\mu_{k}^{i}(t)\right\rceil$ but $\left\lceil 2L_{\text{Data}}^{q}/\mu_{k}^{i}(t)\right\rceil$. The reason is detailed as below: If the state of *L* = *m* has been continued a time of $\left\lceil L_{\text{Data}}^{q}/\mu_{k}^{i}(t)\right\rceil$, it means at that time the node *i* is in the state of sending data but not really sending out. In other words, node *i* makes analysis for its storage state while data is just being sent, which leads the node to make errors because of congestion caused by itself. So, the typical period of large scale fading is $\left\lceil 2L_{\text{Data}}^{q}/\mu_{k}^{i}(t)\right\rceil$.

**Figure 5.**
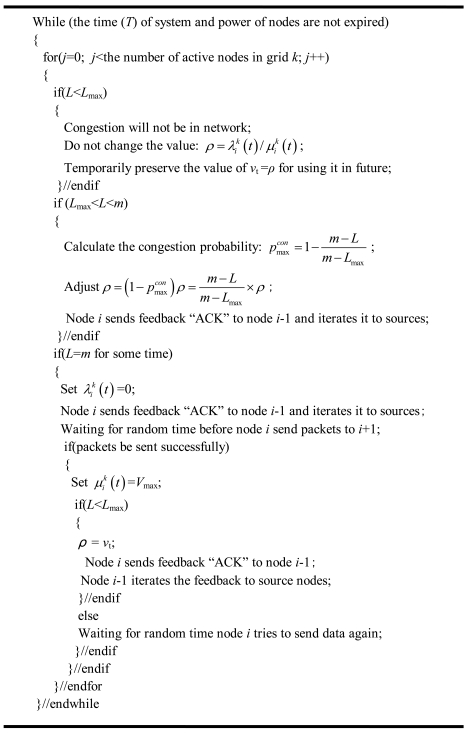
The pseudo-code for single-node rate control method.

In conclusion, the congestion can be effectively unblocked using Equation (7) as well as the ACK messages when the node may experience congestion (the pseudo-code is shown in Figure 5). But all of the above congestion problems only consider the perspective of the node-level. Owing to the occurrence of network congestion with the characteristic of space relevancy, solutions must be obtained to unblock congestion both on the node-level and system-level.

#### 4.1.2. The pre-control and adjustment method of system-level rate

**Figure 6.**
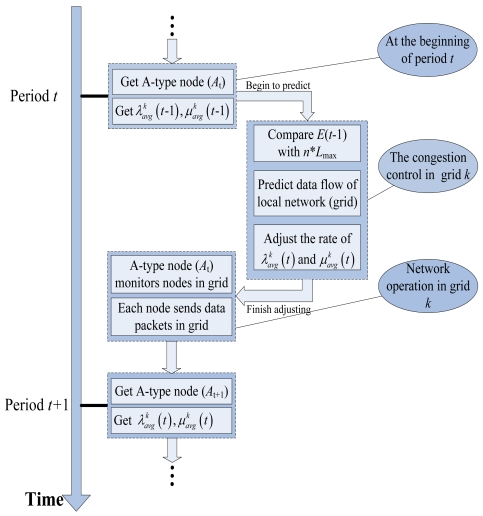
Flow framework of system-level control method.

Now, we describe the system-level congestion control in detail (see Figure 6 for the flow chart of the system-level control method). Node *i* is analyzed in a certain grid *k*. (The grid structure, period and A-type have been described in subsection 3.2). The A-type node has been set on promiscuous mode to record the rate $\lambda_{i}^{k}(t),\mu_{i}^{k}(t)$ of the node *i* in grid *k* for average rate calculation $\lambda_{\text{avg}}^{k}(t),\mu_{\text{avg}}^{k}(t)$. In order to conveniently adjust the average rate according to the local occupied memory resources *E*(*t* – 1), as well as the average inputting and outputting rates in the previous period *t* – 1, CL-APCC predicts the occupied memory resources *E*(*t*) in period *t* and uses the relationship between *E*(*t*) and *n* × *L*_max_, as well as the ratio of *E*(*t*) and *E*(*t* – 1) to decide the adjustment method of the average inputting and outputting rates in period *t*, which avoids the occurrence of local congestion.

At the beginning of the period *t*, it is assumed that the number of nodes to transmit data in grid *k* is *X*(*t*) = *n < N/k* and the average rate is $\lambda_{\text{avg}}^{k}(t)=\sum_{i=1}^{n}\lambda_{i}^{k}(t-1)/n$, $\mu_{\text{avg}}^{k}(t)=\sum_{i=1}^{n}\mu_{i}^{k}(t-1)/n$; if ➀ The probability that data inputted to grid *k* is in direct ratio with Δ*t*, recorded as *b_n_* × Δ*t*, inputting two or more data with a probability that is *O*(Δ*t*), ➁ The probability that data outputted from grid *k* is in direct ratio with Δ*t*, recorded as *d_n_* × Δ*t*, outputting two or more data with a probability that is *O*(Δ*t*). In order to analyze the change of the data quantity in the grid, it first needs to get the mode of *P*_n_(*t*) [41] which means that the event probability can be decompounded as:

- If *X*(*t*) = *n* – 1, input only one quantity of data in the grid within Δ*t*, so the probability is *P*_n-1_(*t*) × *b*_n-1_ ×Δ*t*;
- If *X*(*t*) = *n* + 1, output only one quantity of data in the grid within Δ*t*, so the probability is *P*_n+1_(*t*) × *d*_n+1_ ×Δ*t*;
- If *X*(*t*) = *n*, there is not any inputting and outputting data in the grid within Δ*t*, the quantity of data hasn't changed, so the probability is *P*_n_(*t*) × [1 – *d*_n_ × Δ*t* – *b*_n_ × Δ*t*],

According to the full probability Equation, we can see that:

(8) $$P_{n}(t+\Delta t)=P_{n-1}(t)\times b_{n-1}\times \Delta t+P_{n+1}(t)\times d_{n+1}\times \Delta t+P_{n}(t)\times (1-b_{n}\times \Delta t-d_{n}\times \Delta t)+O(\Delta t)$$

The differential Equation of *P*_n_(*t*) is:

(9) $$\frac{dP_{n}}{dt}=P_{n-1}(t)\times b_{n-1}+P_{n+1}(t)\times d_{n+1}-P_{n}(t)\times (b_{n}+d_{n})$$

It reults that from Equation (9):

(10) $$\frac{dP_{n}}{dt}=P_{n-1}(t)\times \lambda_{\text{avg}}^{k}(t)\times (n-1)+P_{n+1}(t)\times \mu_{\text{avg}}^{k}(t)\times (n+1)-P_{n}(t)\times (\lambda_{\text{avg}}^{k}(t)+\mu_{\text{avg}}^{k}(t))\times n$$

Equation (10) is very complicated and with no easy solution value. Actually, we are only interested in the expectation value *E*(*X*(*t*)) (namely *E*(*t*)) and variance of *D*(*t*). Owing to the fact that *N* can choose any value in theory, in order to conveniently discuss the continuously changing characteristics of the quantity of data, we take the limitation value as the approximately true value that is meaning to assume *n* → +∞. The expected value of data derived from the Equation (10) is:

(11) $$E(t)=\sum_{n=1}^{\infty }n\times P_{n}(t)$$

Educe Equation (11) with Equation (10):

(12) $$\frac{dE}{dt}=\lambda_{\text{avg}}^{k}(t)\sum_{n=1}^{\infty }n(n-1)\times P_{n-1}(t)+\mu_{\text{avg}}^{k}(t)\sum_{n=1}^{\infty }n(n+1)\times P_{n+1}(t)-\left(\lambda_{\text{avg}}^{k}(t)+\mu_{\text{avg}}^{k}(t)\right)\sum_{n=1}^{\infty }n^{2}\times P_{n}(t)$$

Owing to:

(13) $$\begin{array}{c} \sum_{n=1}^{\infty }n(n-1)\times P_{n-1}(t)=\sum_{j=1}^{\infty }j(j+1)\times P_{j}(t)\\ \sum_{n=1}^{\infty }n(n+1)\times P_{n+1}(t)=\sum_{j=1}^{\infty }j(j-1)\times P_{j}(t) \end{array}$$

Substituting Equation (13) to Equation (11) and using Equation (12):

(14) $$\frac{dE}{dt}=\left(\lambda_{\text{avg}}^{k}(t)-\mu_{\text{avg}}^{k}(t)\right)\sum_{n=1}^{\infty }nP_{n}(t)=\left(\lambda_{\text{avg}}^{k}(t)-\mu_{\text{avg}}^{k}(t)\right)E(t)$$

The changing expectation value in the grid derived from Equation (14):

(15) $$E(t)=E(t-1)\times e^{\left(\lambda_{\text{avg}}^{k}(t)-\mu_{\text{avg}}^{k}(t)\right)\times t}$$

The other expression of *E*(*t*) is as below.

(16) $$E(t)=n\times t\times \left(\lambda_{\text{avg}}^{k}(t-1)-\mu_{\text{avg}}^{k}(t-1)\right)\times \frac{e^{\left(\lambda_{\text{avg}}^{k}(t-1)-\mu_{\text{avg}}^{k}(t-1)\right)\times t}}{e^{\left(\lambda_{\text{avg}}^{k}(t-1)-\mu_{\text{avg}}^{k}(t-1)\right)\times t}-1}$$

(proved by Theorem 4 in the Section 5.4)

The changing proportion of storage quantity in the grid is as below:

(17) $$\delta =E(t)/E(t-1)=e^{\left(\lambda_{\text{avg}}^{k}(t)-\mu_{\text{avg}}^{k}(t)\right)\times t}$$

If 0 ≤ *E*(*t*) ≤ *n* × *L*_max_, the average rate keep period *t*-1 unchanged. If *n* × *m* > *E*(*t*) < *n* × *L*_max_, $\lambda_{\text{avg}}^{k}(t)-\mu_{\text{avg}}^{k}(t)$ be decreased as below.

(18) $$\lambda_{\text{avg}}^{k}(t)-\mu_{\text{avg}}^{k}(t)=\left(\lambda_{\text{avg}}^{k}(t-1)-\mu_{\text{avg}}^{k}(t-1)\right)\times \frac{1}{\delta }\left(\sum_{i=1}^{n}\lambda_{i}^{k}(t-1)/n-\sum_{i=1}^{n}\mu_{i}^{k}(t-1)/n\right)\times e^{-\frac{1}{n}\sum_{i=1}^{n}\left(\lambda_{i}^{k}(t-1)-\mu_{i}^{k}(t-1)\right)\times t}$$

**Figure 7.**
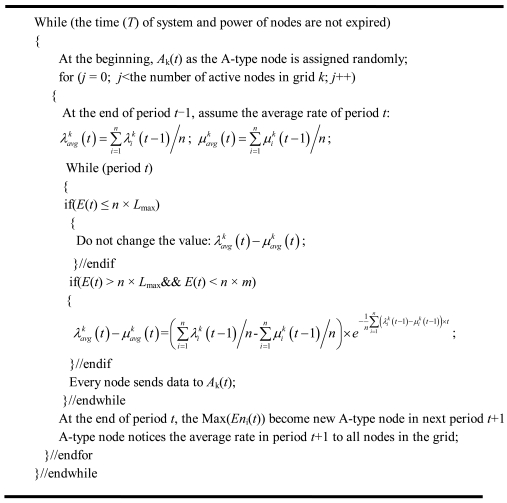
The pseudo-code for local network (grid) rate control method.

It results that the adjustment equation of the average rate in the grid from Equations 17 and 18, which can avoid the local congestion of the network through the adjustment method. The pseudo-code is shown in Figure 7.

#### 4.1.3. The calculation method for real rate of each node

According to subsections 4.1.1 and 4.1.2, it is considered both from the node-level and system-level, which expresses the real rate of the node as below.

The inputting rate of node in period *t*.

(19) $$\lambda_{\text{real},i}^{k}(t)=\alpha \lambda_{\text{avg}}^{k}(t)+(1-\alpha )\lambda_{i}^{k}(t)$$

The outputting rate of node in period *t*.

(20) $$\mu_{\text{real},i}^{k}(t)=\alpha \mu_{\text{avg}}^{k}(t)+(1-\alpha )\mu_{i}^{k}(t)$$

In sum, the pre-control rate of the node-level is combined with that of the system-level to control congestion, which takes into account the relationship between one part and the whole of the network. In the practical application, CL-APCC protocol increases the value of weight *α* when bandwidth and channel quality is in the higher proportion to effectively use the network resources in the whole. Whereas, CL-APCC protocol decreases the value of weight *α* when the retransmission quantity of a certain node is lower and its child-node is less, which is aimed to make the node effectively use its own resources (such as storage space and rate, etc.) to obtain the highest efficiency of the network in the whole.

### 4.2. The Revised IEEE 802.11 Protocol of CL-APCC

In this section, the IEEE 802.11 protocol is revised in order to decrease the transmission conflicts of data packets and ensure the fairness and timeliness of networks. In the network, there are different requirements of multi-data flow on reliability and sending timeliness, so it is necessary to have a priority control strategy in WSNs. In this paper, according to the sending priority and he service time of packets at the node, CL-APCC protocol makes a rule on how to deal with the priority of data packets.

First, the revised IEEE 802.11 protocol method is described in general. The CL-APCC protocol dynamically adjusts the current competition window (CW) of a node based on the original priority and the service time of packets at the node. As the times of node's competition channel increase, the size of CW gradually decreases and accordingly results in an increase in the probability which acquires the current slot. Once the node competes to obtain slot in period *t*, then its competition probability reduces to the minimum (CW = *β*CW_max_) till the end of the period *t*.

Next, we describe the revised method of the IEEE 802.11 protocol in detail as follows:

Node *i* is analyzed in a certain grid *k*. Assume the waiting time is ${\text{W}}_{i}^{q}(t_{1}t_{2})$ which means packet *q* service time at the node *i*, the length of packet *q* is $L_{\text{Data}}^{q}$, the original priority of packet *q* is $p_{\text{initial}}^{q}\;(t)$. So the sending priority of this packet is calculated as ${\text{p}}_{i}^{q}(t)=p_{\text{initial}}^{q}(t)\times {\text{W}}_{i}^{q}(t_{1}t_{2})$. According to the feature of sending priority of packets, node *i* takes $\text{Max}\left({\text{p}}_{i}^{q}(t)\right)$ as a packet to be sent. At the beginning of period *t*, node *i* sends the value of $\text{Max}\left({\text{p}}_{i}^{q}(t)\right)$ to the A-type node in the grid. After the integrated calculation of the A-type node, it can be obtained the sending probability of node *i* in the first slot as Equation (21). Concurrently, the A-type node notices the sending sequence of nodes to all nodes in the grid. Furthermore, each node maintains a counter to remember the sequence for the calculation of its sending probability and to deal with A-type node failure (as described in subsection 3.2).

(21) $$R_{i}(t)=\frac{{\text{p}}_{i}^{q}(t)\times {\text{W}}_{i}^{q}(t_{1},t_{2})}{\sum_{i=1}^{n}{\text{p}}_{i}^{q}(t)\times {\text{W}}_{i}^{q}(t_{1},t_{2})}$$

It will be divided into two situations to discuss the sending probability of node *i*: ➀ if node *i* can compete to obtain a sending slot at its own moment, then *R_i_*(*t*) = 0, the other nodes in the grid will switch into the sleeping state in the next $\left\lceil L_{\text{Data}}^{q}/\mu_{\text{real},i}(t)\right\rceil$ slot to save energy. When node *i* finishes sending a data packet, the other nodes in the grid begin to compete for the channel. ➁ If node *i* doesn't compete to obtain the channel at the moment, it competes again after the node which has already competed to obtain slots and finished sending data. The sending probability of node *i* is updated as below:

(22) $$R_{i}(t)=R_{i}(t)+J\times (1-R_{i}(t))/n_{\text{con}}^{i}$$

**Figure 8.**
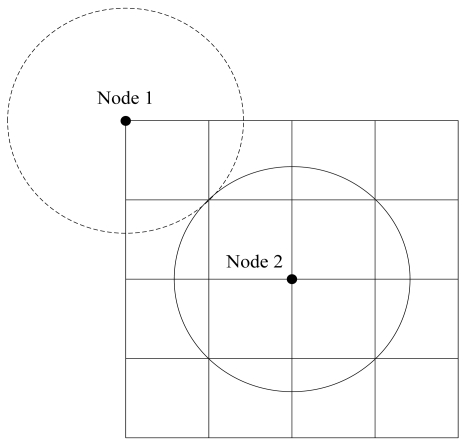
Neighbors in the communication range of one node.

*J* is competition times and $n_{\text{con}}^{i}$ is neighbor quantity in the communication range of node *i* (each node's value of $n_{\text{con}}^{i}$ is different), it can show the scope of $n_{\text{con}}^{i}$ in Figure 8.

(23) $$(N\times \pi \times r^{2})/(4\times L^{2})\leq n_{\text{con}}^{i}\leq (N\times \pi \times r^{2})/L^{2}$$

**Figure 9.**
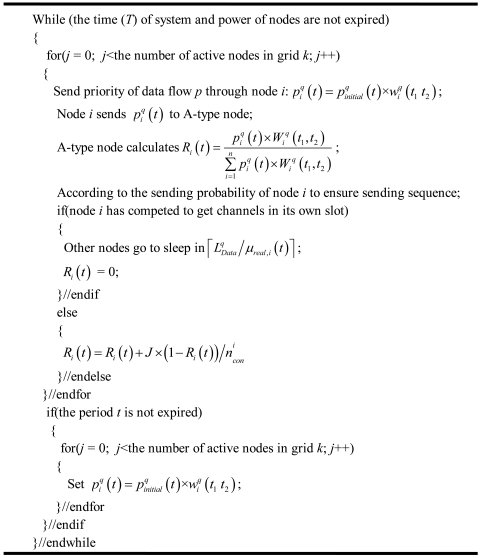
The pseudo-code for the revised IEEE 802.11 protocol.

At the end of the period *t*, the A-type node in the grid turns to the next period of calculation for the average rate and node sending priority. The pseudo-code is shown in Figure 9.

## 5. Performance Analysis of the CL-APCC Protocol

In this section, we would like to analyze each performance feature of the CL-APCC protocol, such as time complexity, control complexity, energy complexity, as well as storage overhead.

### 5.1. Time Complexity Analysis of CL-APCC Protocol

#### Theorem 1

In any grid, the time complexity of period *t* is *O*(*N*), the time complexity is *O*(*fN*) which is from data generated to be sent to BS, and *f* is the biggest hop of network routing.

#### Proof

The period *t* of each grid is decided by the situation of the local network, the sending rate of each node, the length of data flow and the conflict regions of each node. Node *i* is analyzed in a certain grid *k*. When the period *t* is at the maximum, the sending conflict times of each node in the grid is also at the maximum. So, it is necessary that the sending probability is 1 when node *i* finally sends a data packet, it's available by Equation (24):

(24) $$R_{i}(t)+J\times (1-R_{i}(t))/n_{\text{con}}^{i}=1\Rightarrow J=n_{\text{con}}^{i}$$

From Equations (23) and (24) we can see the maximum conflict times of node *i* is as below:

(25) $$\text{Max}(J)=(N\times \pi \times r^{2})/L^{2}$$

If we know the existing waiting time it takes node *i* to send data, we can obtain the complexity equation of the period *t*. In the conflict region of node *i*, the time length of packet sending for each node is $\left\lceil L_{\text{Data}}^{q}/\mu_{\text{con}}^{i}(t)\right\rceil$. The packet length $L_{\text{Data}}^{q}$ is fixed, so if it makes $\left\lceil L_{\text{Data}}^{q}/\mu_{\text{con}}^{i}(t)\right\rceil$ maximal, then $\mu_{\text{con}}^{i}(t)$ needs to be minimal. Assume that the data inputting rate $\lambda_{\text{con}}^{i}(t)$ which is generated by data sources is already known. From the Queuing theory (subsection 4.1.1), $0\leq \rho =\lambda_{i}^{k}(t)/\mu_{i}^{k}(t)\leq 1$, it can be obtained $\text{Min}\left(\mu_{\text{con}}^{i}(t)\right)=\lambda_{i}^{k}(t)$, so the longest time of period *t* is attained by the Equation (22):

(26) $$\text{Max}(t)=\text{Max}(J)\times L_{\text{Data}}^{q}/\text{Min}\left(\mu_{\text{con}}^{i}(t)\right)=\frac{N\times \pi \times r^{2}\times L_{\text{Data}}^{q}}{\text{Min}\left(\mu_{\text{con}}^{i}(t)\right)}$$

So the time complexity of arbitrary grid complexity is *O*(*N*). Similarly, the maximum time of data from generated until being sent to BS is shown as below:

(27) $$\text{Max}(i_{t})=\frac{f\times N\times \pi \times r^{2}\times L_{\text{Data}}^{q}}{\text{Min}\left(\mu_{\text{con}}^{i}(t)\right)}$$

Its time complexity is *O* (*fN*). Therefore, the theorem 1 is proved to be correct.

### 5.2. Control Complexity Analysis of CL-APCC Protocol

#### Theorem 2

It is *O*(*N*) that is the complexity of control message in the whole network.

#### Proof

The majority of control information is carried out incidentally at packet header. It is divided into two kinds of control information. ➀ During the network initialization, each node monitors the notice information of its neighbors to obtain its own conflict region. The monitoring process requires each node to send data once. The complexity is *O*(*N*) for this kind of control information. ➁ The A-type node of each grid at the end of period *t* broadcasts information about the next period, including the average rate of the grid, the sending probability of each node, and the new A-type node information. The complexity is *O*(*K*) for this kind of control information.

To sum up the complexity of the two kinds of control information, it can be seen that *O*(*N*) is the control complexity of the network. Therefore, the theorem 2 is proved to be correct.

### 5.3. Energy Complexity Analysis of CL-APCC Protocol

#### Theorem 3

*O*(*N*^2^/*K*) is the complexity of energy in any grid, it is further known that *O*(*N*^2^) is the energy overhead of the whole network.

#### Proof

It is mainly divided into four parts for the whole network's energy overhead which are data sending, data receiving, node monitoring and node calculation. Owing to the fact that the energy overhead of node monitoring and calculation is much smaller than that of data sending and receiving, we only take into account the energy overhead of data sending and receiving.

From figure 3, the outputting rate of *i* – 1 is equal to the inputting rate of *i*. If it gets the data rate generated by data sources, in accordance with the relationship between the average rates of inputting and outputting in section 4.1.2, we can analyze the average energy overhead of the grid in the network. Using the energy overhead model of the literature [20], the energy overhead is *E_TX_* = *E_elec_* × *L* + *ε_fs_* × *L* × *d*^2^ which is transmitted by node from data packets with the length of *L* to the distance of *d*, and energy overhead of data receiving is *E_RX_* = *E_elec_* × *L*. Thus, for one grid, data transmission of energy overhead within period *t* is shown as below:

(28) $$\begin{array}{c} E_{TX}^{k}(t)=n\times t\times E_{TX}\times \mu_{\text{avg}}^{k}(t)=n\times t\times (E_{\text{elec}}\times L+\varepsilon_{fs}\times L\times d^{2})\times \mu_{\text{avg}}^{k}(t)\\ E_{RX}^{k}(t)=n\times t\times E_{RX}\times \lambda_{\text{avg}}^{k}(t)=n\times t\times E_{\text{elec}}\times L\times \lambda_{\text{avg}}^{k}(t) \end{array}$$

The total energy overhead of the grid within period *t* is as below:

(29) $$E_{\text{total}}^{k}(t)=E_{TX}^{k}(t)+E_{RX}^{k}(t)=n\times t\times (E_{\text{elec}}\times L+\varepsilon_{fs}\times L\times d^{2})\times \mu_{\text{avg}}^{k}(t)+n\times t\times E_{\text{elec}}\times L\times \lambda_{\text{avg}}^{k}(t)$$

From the Equation (29) and *n* < *N/K*, we know that:

(30) $$\text{Max}\left(E_{\text{total}}^{k}(t)\right)=\frac{N^{2}\times \pi \times r^{2}\times L_{\text{Data}}^{q}}{K\times \text{Min}\left(\mu_{\text{con}}^{i}(t)\right)}\times \left(\left(E_{\text{elec}}\times L+\varepsilon_{fs}\times L\times d^{2})\times \mu_{\text{avg}}^{k}(t)+E_{\text{elec}}\times L\times \lambda_{\text{avg}}^{k}(t\right)\right)$$

Owing to *d* ≤ *r*, *O*(*N*^2^/*K*) is the complexity of energy overhead in the grid, it is further known that *O*(*N*^2^) is the complexity of energy overhead in the whole network. Therefore, theorem 3 is proved to be correct.

### 5.4. Storage Overhead Analysis of CL-APCC Protocol

#### Theorem 4

The storage overhead of a single-node is $\frac{\rho^{2}[1-m\times \rho^{m-1}(m-1)\times \rho ]}{\left(1-\rho )\times (1-\rho^{m+1}\right)}$, the storage overhead of a grid is $\frac{n\times t\times \left(\lambda_{\text{avg}}^{k}(t-1)-\mu_{\text{avg}}^{k}(t-1)\right)}{e^{\left(\lambda_{\text{avg}}^{k}(t-1)-\mu_{\text{avg}}^{k}(t-1)\right)\times t}-1}\times e^{\frac{\left(\lambda_{\text{avg}}^{k}(t-1)-\mu_{\text{avg}}^{k}(t-1)\right)\times t}{e^{\left(\lambda_{\text{avg}}^{k}(t-1)-\mu_{\text{avg}}^{k}(t-1)\right)\times t}}}$, and the storage overhead of the whole network is $k\times \frac{n\times t\times \left(\lambda_{\text{avg}}^{k}(t-1)-\mu_{\text{avg}}^{k}(t-1)\right)}{e^{\left(\lambda_{\text{avg}}^{k}(t-1)-\mu_{\text{avg}}^{k}(t-1)\right)\times t}-1}\times e^{\frac{\left(\lambda_{\text{avg}}^{k}(t-1)-\mu_{\text{avg}}^{k}(t-1)\right)\times t}{e^{\left(\lambda_{\text{avg}}^{k}(t-1)-\mu_{\text{avg}}^{k}(t-1)\right)\times t}}}$.

#### Proof

(1) First of all, the storage overhead of a single-node is to be analyzed. In subsection 4.1.1, we can see that the analysis focused on a single-node should consider not only the optimal region but the whole memory size of the node. Therefore, it is feasible to use the mixed queuing mode M/M/1/m to analyze the average length of waiting queue in the node, and *L_q_* is shown as below.

(31) $$L_{q}=\rho^{2}\times p_{0}\times \sum_{n=1}^{m-1}\left(n\times \rho^{n-1}\right)=\frac{\rho^{2}[1-m\times \rho^{m-1}(m-1)\times \rho ]}{\left(1-\rho )\times (1-\rho^{m+1}\right)}$$

(2) Take grid *k* for analysis of storage overhead. At the beginning of period *t*, if it is not adjusted for the average rate of the period *t* – 1, and the average storage overhead of data in the grid is shown as below.

(32) $$E(t)=E(t-1)+n\times t\times \left(\lambda_{\text{avg}}^{k}(t-1)-\mu_{\text{avg}}^{k}(t-1)\right)$$

It is available by the Equation (15) and (32).

(33) $$\begin{array}{c} E(t-1)\times e^{\left(\lambda_{\text{avg}}^{k}(t)-\mu_{\text{avg}}^{k}(t)\right)\times t}=E(t-1)+n\times t\times \left(\lambda_{\text{avg}}^{k}(t-1)-\mu_{\text{avg}}^{k}(t-1)\right)\\ E(t-1)=\frac{n\times t\times \left(\lambda_{\text{avg}}^{k}(t-1)-\mu_{\text{avg}}^{k}(t-1)\right)}{e^{\left(\lambda_{\text{avg}}^{k}(t-1)-\mu_{\text{avg}}^{k}(t-1)\right)\times t}-1} \end{array}$$

Therefore, the Equation (16) in Section 4.1.2 is proved to be correct. Whilst, *E*(*t* – 1) is the storage overhead of grid *k* at the beginning of period *t*. Next, the average storage overhead of the grid is calculated in period *t*. It is derived from prediction that the relationship between *E*(t) and *n* × *L*_max_, combined with the Equation (16), that the real storage overhead within period *t* in the grid is as follows.

(34) $$E_{\text{real}}(t)=E(t-1)\times e^{\frac{\left(\lambda_{\text{avg}}^{k}(t-1)-\mu_{\text{avg}}^{k}(t-1)\right)\times t}{\delta }}$$

It is available by the Equations (15), (17), (33) and (34)

(35) $$E_{\text{real}}(t)=\frac{n\times t\times \left(\lambda_{\text{avg}}^{k}(t-1)-\mu_{\text{avg}}^{k}(t-1)\right)}{e^{\left(\lambda_{\text{avg}}^{k}(t-1)-\mu_{\text{avg}}^{k}(t-1)\right)\times t}-1}\times e^{\frac{\left(\lambda_{\text{avg}}^{k}(t-1)-\mu_{\text{avg}}^{k}(t-1)\right)\times t}{e^{\left(\lambda_{\text{avg}}^{k}(t-1)-\mu_{\text{avg}}^{k}(t-1)\right)\times t}}}$$

(3) Within one period, the storage overhead of the whole network equals to the product. This means the storage overhead of each grid multiplies the number of grids, *K*. Therefore, the overall storage overhead of network *M_total_*(*t*) is shown as follows.

(36) $$M_{\text{total}}(t)=K\times E_{\text{real}}(t)=K\times \frac{n\times t\times \left(\lambda_{\text{avg}}^{k}(t-1)-\mu_{\text{avg}}^{k}(t-1)\right)}{e^{\left(\lambda_{\text{avg}}^{k}(t-1)-\mu_{\text{avg}}^{k}(t-1)\right)\times t}-1}\times e^{\frac{\left(\lambda_{\text{avg}}^{k}(t-1)-\mu_{\text{avg}}^{k}(t-1)\right)\times t}{e^{\left(\lambda_{\text{avg}}^{k}(t-1)-\mu_{\text{avg}}^{k}(t-1)\right)\times t}}}$$

Therefore, the theorem 4 is proved to be correct.

## 6. Performance Evaluation

In this section, we present the results of several simulations to evaluate the performance of our congestion control strategies. Our simulations focus on the RPR of network under different conditions. We simulate the proposed scheme on VC++. The simulations are divided into three parts. (1) Simulation analyses for the various performances of CL-APCC protocol. (2) Simulation analyses about the impact of the revised IEEE 802.11 protocol for the network.(3) CL-APCC protocol is compared with other congestion control protocols.

**Figure 10.**
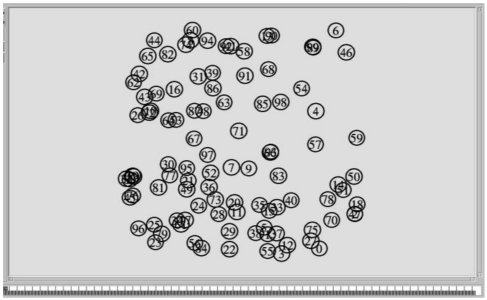
Random distribution map of 100 nodes.

Here, the CL-APCC protocol develops a simulation environment which is based on the following models. The dimension of area monitoring is 100 m × 100 m, nodes are randomly arranged (Figure 10), the physical layer adopts double-track model and the ideal channel (no large-scale decline). The MAC layer uses the IEEE 802.11 protocol. CL-APCC uses the energy model of sending and receiving and the communication radius of nodes in literature [42]. The initial energy of the node is 2 J. It takes 200 seconds for each simulation, At the same time, we set the initial sending rate for the node of data sources based on the data requirement of BS, and the initial RPR is set to be the average value of the whole network's RPR in the time of 200 seconds. The route protocol is set as AODV and the communication radius is *r* = 40 m. Moreover, some of the parameters values are changed for the scenes and experimental goals that have changed. During the process of stimulation, unless specified, we make a data sampling every 40S and obtain the data statistics which are used to describe the performance of CL-APCC. All the parameters used in the simulations are shown in Table 1.

### 6.1. Performance Analysis of CL-APCC

To evaluate the performance of the proposed CL-APCC scheme, we make some simulations as below.

#### 6.1.1. The universal performance of CL-APCC

**Figure 11.**
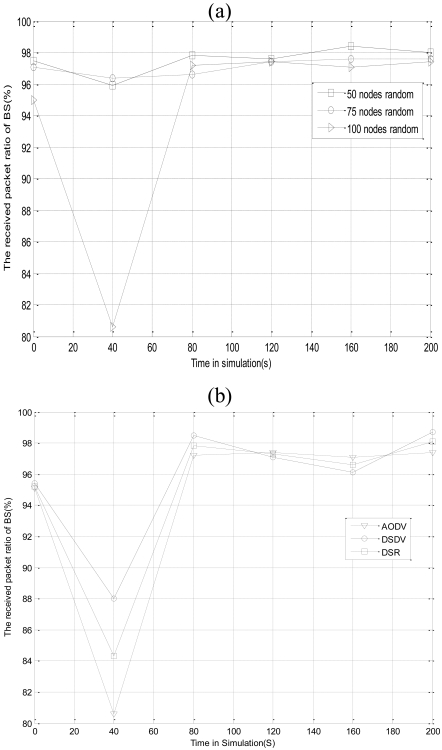
(a). Different node density on the impact for the RPR of BS; (b). Different routers on the impact for the RPR of BS.

Most existing congestion control algorithms only act directly against specific network density and routing protocols. In order to illustrate the universal feature of the proposed scheme, in the first set of simulations, we describe the impact of different network densities and routings for the RPR of network in Figure 11.

In Figure 11(a), when the node number is 50, the average RPR of BS is 97.5%. In the same way, when the node number is 75, the average RPR of BS is 97.1%, and when the node number is 100, the average RPR of BS is 95.1%. It can be seen that admission ratio of BS has a small extent of decline with the number of nodes increased. That is because data packets generated by the network correspondingly increase with the number of node increased. Consequently, there is a high probability of data sending collisions between nodes, and all the factors mentioned above result in the increase of lost packets ratio. However, the network has a stable trend, and after 40 seconds the PRP of the network is stabilized from 95% to 97%. The average RPR of network stays above 95%, which can better meet the goal of network monitoring. Now, we analyze the reason that the RPR of the network is lower in the previous 40 seconds. When the node number is 100, the average admission ratio in the pre-40s of the experiment is only 80.6%. This is because at the initial time, data packets are temporarily cached in some nodes, which results in data packets which are generated by data sources not arriving at BS in time. As time goes by, CL-APCC adjusts the data sending rate of the node based on the memory conditions of the node which makes the RPR of the network achieve balance.

In Figure 11(b), the node number is 100. The network trends to stable after 40 seconds. It is less than 1% the impact to CL-APCC, which is caused by the different routing protocols. The RPR of the network is kept balanced at 97%. Everything mentioned above illuminate that CL-APCC is irrelevant to the specific routing protocol. It has the higher universal feature. Furthermore, the reason that the RPR is smaller in the pre-40s of the network is the same as Figure 11 (a).

#### 6.1.2. The average queue length of node

The average queue length (AQL) of a node reflects the relationship between the inputting and outputting rate of the node, which affects the time from the data being generated until it is sent to BS. As the network becomes stable after 40 seconds, we make a sampling every 5 seconds on the queue length of each node between 60 seconds and 150 seconds to obtain statistical data by random sampling nodes. Then the average queue lengths of nodes are calculated with different networks densities.

**Figure 12.**
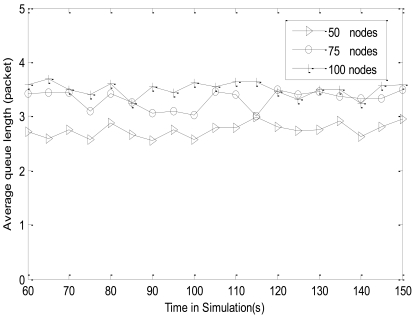
The average queue length of node in the network.

Figure 12 shows that AQL keeps around half of the total memory of the node (3 packets), which shows that the network is in a stable state. Whilst, AQL is less than *L*_max_ (*L*_max_ = 3.75 packets), which is due to the fact that CL-APCC protocol is able to adjust the sending rate of the node when the occupied memory size (*L*) is more than *L*_max_, This further avoids network congestion.

#### 6.1.3. The average energy consumption of each data packet

The average energy consumption (AEC) of data packets received by BS reflects the length of the network's lifetime. The routing protocol is set as AODV and the number of nodes is 100. We respectively obtain the statistical data by random sampling nodes for the network with CL-APCC and without CL-APCC. AEC is calculated as Equation (37):

(37) $$\begin{array}{c} E_{Tx}(L_{\text{Data}})=E_{\text{elec}}\times L_{\text{Data}}+{ɛ}_{\text{amp}}\times L_{\text{Data}}\times d^{2}\\ E_{Rx}(L_{\text{Data}})=E_{\text{elec}}\times L_{\text{Data}}\\ n_{\text{average}\_\text{hop}}=\frac{\sum_{i=1}^{D_{\text{total}}}n_{i\_\text{hop}}}{D_{\text{total}}}\\ E_{\text{average}}=E_{Tx}(L_{\text{Data}})\times n_{\text{average}\_\text{hop}}+E_{Rx}(L_{\text{Data}})\times \left(n_{\text{average}\_\text{hop}}-1\right) \end{array}$$

The parameters in the Equation (37) are described as follows. *E_Tx_*(*L_Data_*) is the sending energy consumption of data packets, *E_Rx_*(*L_Data_*) is the receiving energy consumption of data packets, *n_average_hop_* is the average hops of data packets, and *E_average_* is the average energy consumption.

**Figure 13.**
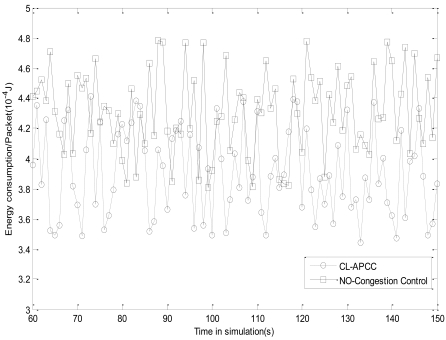
The average energy consumption of each data packets received by BS.

Figure 13 shows that the average energy consumption of packet with CL-APCC is less than without CL-APCC. That is because CL-APCC is so effective at solving network congestion and avoiding the repeated sending of data packets, which can reduce the energy consumption of the whole network and increase the lifetime of WSNs.

#### 6.1.4. Communication radius *r* on the impact for CL-APCC protocol

The size and number of the grids are decided by communication radius *r*, which affects the control effect of CL-APCC on network congestion. Hence, it needs to make stimulations on the impact for the RPR of CL-APCC with the different network densities and communication radiuses. We respectively set the communication radius to *r* = 40 m and 50 m, and the number of nodes is 50, 75 and 100.

**Figure 14.**
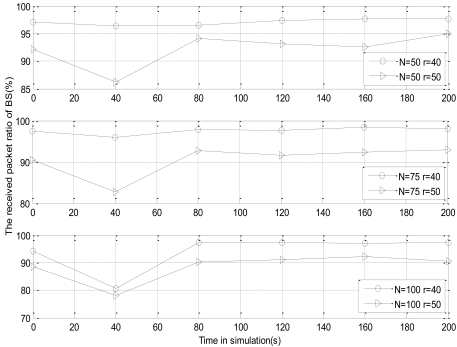
Different communication radius on the impact for CL-APCC.

Figure 14 shows that the RPR of the network declines when the communication radius is increased. The reason is that ➀ the bigger the communication radius is, the greater the number of the neighbor nodes is, so the possibilities of data sending collisions between the nodes are correspondingly increased. ➁The communication radius of the node has an impact on the size of the grid. That is to say, if the communication radius of the node increases, the number of grids decreases, and then the local control capacity of CL-APCC on the network will accordingly decline. Therefore, the overall RPR of the network also declines.

### 6.2. The Performance of the Revised IEEE 802.11 Protocol

To evaluate the performance of the revised IEEE 802.11 protocol, we make some simulations on the revised 802.11 protocol against the IEEE 802.11 protocol, including the number of average collisions in data sending, the RPR of the network, and the priority of data packets.

#### 6.2.1. The number of average collisions

**Figure 15.**
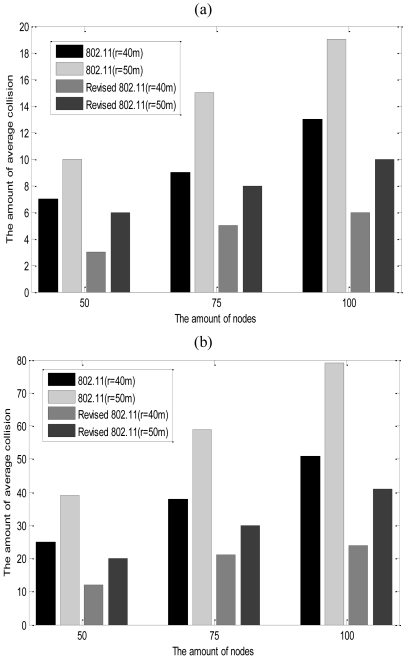
(a) The minimal AAC in the network; (b) The maximal AAC in the network.

The energy overhead of the network and the timeliness of data packets are affected by the amount of average collisions(AAC) of nodes. Hence, we make analyses on the maximum and minimum AAC of CL-APCC. The AAC of IEEE 802.11 protocol is $n_{\text{con}}^{i}$, and the AAC of the revised IEEE 802.11 protocol is calculated by the Equation (38). The communication radius is *r* = 40 m or 50 m. The simulation results are shown in Figure 15.

(38) $$\frac{\sum_{k=1}^{n_{\text{con}}^{i}}\left(\frac{\pi r^{2}}{R^{2}}\times N-k\right)}{n_{\text{con}}^{i}}$$

Figure 15 shows that the AAC of the revised IEEE 802.11 protocol is only 50% that of the IEEE 802.11 protocol. It is because CL-APCC adopts the way of gradually increasing the probability that a node competes to obtain the current channel, which greatly reduces the number of average collisions, which in turn prolongs the lifetime of the network and increases the timeliness of data packets.

#### 6.2.2. The RPR of network

**Figure 16.**
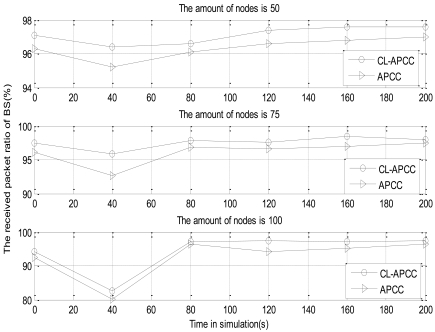
The impact of revised IEEE 802.11 protocol for the RPR of the network.

In order to analyze the impact of the revised IEEE 802.11 protocol on the RPR of the network, we separately simulate on CL-APCC and APCC, which do not use cross-layer optimization. The simulation results are shown in Figure 16.

Figure 16 shows that there is not much difference between the two algorithms on the RPR of the network. CL-APCC is merely 1.2% better than APCC. In other words, the advantage of CL-APCC is not obvious with the RPR of the network. The reason for presenting the cross-layer optimization in CL-APCC is mainly to reduce the energy overhead and ensure the fairness of the network. It solves the RPR of the network with the use of node-level and system-level congestion control strategies in APCC.

#### 6.2.3. The fairness of network

**Figure 17.**
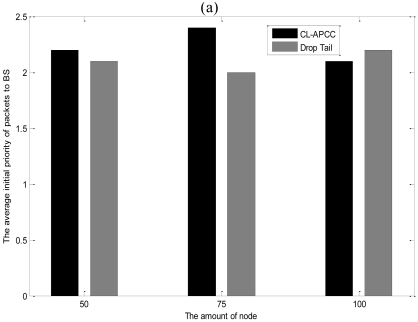

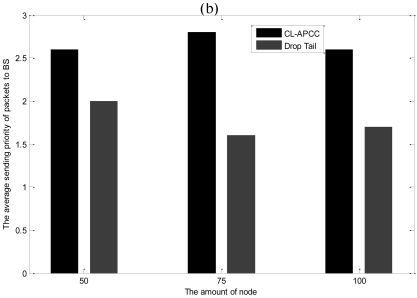
(a) The average initial priority of packets to BS; (b) The average sending priority of packets to BS.

The existing algorithms do not integrate with the issue of congestion and fairness of network in Section 2. In order to illustrate the revised IEEE 802.11protocol on the fairness of network, we adopt “CL-APCC” and “Drop Tail” strategy by random sampling nodes to make simulations. During the simulations, the original priorities of data packets in the data source node are randomly set to 1, 2, and 3. The results are shown in Figure 17.

Figure 17(a) shows that the advantage of CL-APCC is not particularly evident in regard to the average priority of data packets received by BS. However, it can be seen in Figure 17(b) that the average sending priority of data packets with CL-APCC is much higher than ‘Drop Tail’, and the largest gap is 1.3 (the average sending priority is only 2). That is because CL-APCC considers not only the original priority of data packets but the timeliness of the network in the course of sending packets. As described in Section 4.2, in CL-APCC, the sending priority of data packets is correspondingly improved as the service time at the node increases, which ensures the fairness of network.

### 6.3. Compared CL-APCC with Other Congestion Control Protocols

**Figure 18.**
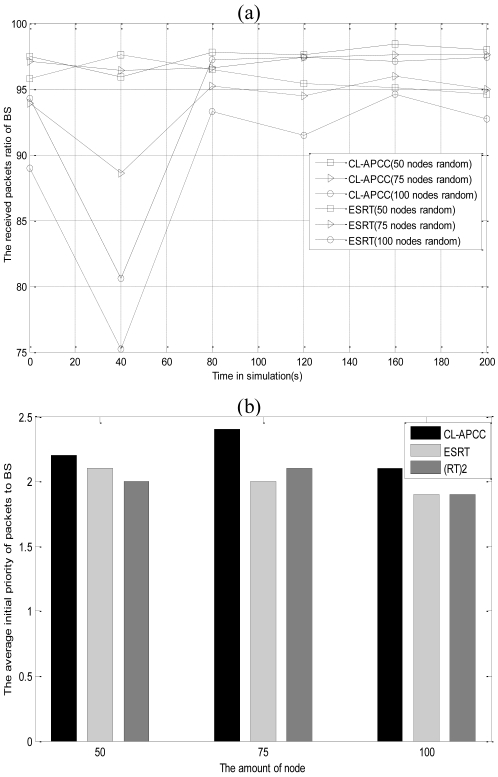
(a) CL-APCC in comparison with the ESRT;. (b) CL-APCC in comparison with the ESRT and (RT)^2^.

In this section, we compare CL-APCC with other congestion control methods for fairness and the RPR of networks in order to illustrate the advantage of CL-APCC protocol. It is separately compared with the typical protocols of ESRT and (RT)^2^ which are similar to the design concept of CL-APCC. The simulation results are shown in Figure 18.

Figure 18(a) shows that the RPR of CL-APCC protocol is higher than that of ESRT protocol, with different time and different number of nodes. If the node number is 100, the average admission packet ratio of CL-APCC is 4.3% higher than ESRT. It shows that CL-APCC applies not only to the low-density network but the high-density network, which demonstrates that CL-APCC is more versatile than ESRT. From Figure 18(b) we can see that the average priority of data packets received by BS is the maximum with the use of CL-APCC protocol. It is because that under the premise of ensuring the timeliness of data packets while CL-APCC is using dynamic priority, the priority of data packets is higher. As such, it is easier to obtain the network resource. Everything mentioned above ensures the fairness of the network.

## 7. Conclusions

Congestion has a severe influence on network performance, which results in a large number of missing packets, unfair status of network and significant wasted energy due to the repeated sending packets. Focusing on the problems, we propose a cross-layer active predictive congestion control protocol (CL-APCC) for WSNs. The basic concept of the CL-APCC protocol is described as below.

Each node adopts a single service window of mixed queuing model M/M/1/m to predict and resolve node-level congestion. CL-APCC predicts the probability of congestion caused by the grid in period *t*, and the probability is used to control the inputting and outputting rate of the grid, which effectively solves the system-level congestion problems of the network. Therefore, the congestion is solved through the combination of node-level control method and system-level control method. At the same time, the IEEE 802.11 protocol is revised in CL-APCC to meet the requirement of fairness of networks, which is depending on the service time at the node and original priority of data packets. We make a complete analysis and simulate on the proposed scheme, which shows that it exceeds other methods relying on not only RPR and lifetime of network but fairness and timeliness of data packets.

However, the kind of congestion control protocols under the circumstances where nodes can be moved have not been considered. Still, we have not yet found an optimal method to set communication radius for the node. Nevertheless, such issues are to be studied in the future so that CL-APCC protocol has much wider applications.

## Figures and Tables

**Table 1.** The setting of parameters in the simulation.

**Parameter**	**Values**
NODE-PLACEMENT	Random
PROPAGATION-PATHLOSS	Two-Ray
PACKET-SIZE	Packet Head Size 12 byte
	Packet Data Size 500 byte
ROUTER-PROTOCOL	ADOV
MAC-PROTOCOL	The IEEE 802.11 Protocol
INITIAL-ENERGY	2 J
Eelec	50 nJ/bit
ξamp	100 pJ/bit/m^2^
TANSMISSION-RANGE (*r*)	40 m
GRIP-NUMBER (*K*)	4 × 4
BS-POSITION	Left-Upper of Monitor Region
NODE-NUMBER (*N*)	50, 75, 100
NETWORK-SIZE	100 m × 100 m
MEMORY-SIZE (*m*)	3 M
ORIGINAL-PRIORITY OF PACKET	1, 2, 3
THRESHOLD (*L*_max_)	0.75 × 3 M
INITIAL *ρ*	0.6
SIMULATION-TIME	200 S
SAMPLING-INTERVAL	5 S, 40 S
